# Usefulness of a Multicomponent Group Intervention Program for Fibromyalgia Patients in Primary Care: A Qualitative Study of Health Professionals

**DOI:** 10.3390/healthcare12010017

**Published:** 2023-12-20

**Authors:** Rosa Caballol Angelats, Carina Aguilar Martín, Noèlia Carrasco-Querol, Rosa Abellana, Gemma González Serra, Dolors Gràcia Benaiges, Maria Cinta Sancho Sol, Immaculada Fusté Anguera, Susana Chavarria Jordana, Anna Berenguera, Alessandra Queiroga Gonçalves

**Affiliations:** 1 Equip d’Atenció Primària Tortosa Est, Institut Català de la Salut, 43500 Tortosa, Spain; 2 Unitat d’Expertesa en Síndromes de Sensibilització Central Terres de l’Ebre, Institut Català de la Salut, 43500 Tortosa, Spain; 3 Programa de Doctorat Medicina i Recerca Translacional, Departament de Fonaments Clínics, Facultat de Medicina, Universitat de Barcelona, 08036 Barcelona, Spain; 4 Unitat de Suport a la Recerca Terres de l’Ebre, Fundació Institut Universitari per a la Recerca a l’Atenció Primària de Salut Jordi Gol i Gurina (IDIAPJGol), 43500 Tortosa, Spain; 5 Unitat d’Avaluació, Direcció d’Atenció Primària Terres de l’Ebre, Institut Català de la Salut, 43500 Tortosa, Spain; 6 Departament de Fonaments Clínics, Facultat de Medicina, Universitat de Barcelona, 08036 Barcelona, Spain; 7 Servei de Rehabilitació i Medicina Física, Hospital de Tortosa Verge de la Cinta, Gerència Territorial de Terres de l’Ebre, Institut Català de la Salut (ICS), 43500 Tortosa, Spain; 8 Centre de Salut Mental d’Adults de Fundació Pere Mata Terres de l’Ebre, 43500 Tortosa, Spain; 9 Equip d’Atenció Primària l’Ametlla de Mar—El Perelló, Institut Català de la Salut, 43860 L’Ametlla de Mar, Spain; 10 Unitat Transversal de Recerca, Fundació Institut Universitari per a la Recerca a l’Atenció Primària de Salut Jordi Gol i Gurina (IDIAPJGol), 08007 Barcelona, Spain; 11 Departament d’Infermeria, Universitat de Girona, 17003 Girona, Spain; 12 Red de Investigación en Cronicidad, Atención Primaria y Promoción de la Salud (RICAPPS), 28041 Madrid, Spain

**Keywords:** fibromyalgia, health personnel, qualitative research, physical therapy, psychology, primary healthcare

## Abstract

Objective: To explore the perceptions and experiences of health professionals who participated in a multicomponent program for fibromyalgia (FM) patients based on health education, physical exercise and cognitive–behavioral therapy. Methods: In this qualitative, descriptive study that was based on a pragmatic and utilitarian approach, we conducted two focus groups (FGs) with 12 professionals (nurses and general practitioners) from the primary healthcare system of Spain who had been trained as FM experts. A thematic content analysis was carried out. Results: The findings were organized into four key domains, each with explanatory emerging themes. Overall, the professionals positively valued the program for the knowledge gain it offered, its integrated approach, the group effect and other benefits to patients. Work overload and peers’ lack of acknowledgement of the program’s value were identified as barriers. A reduction in the amount of content in each session, the creation of quality-of-care indicators and the promotion of the new professional role (FM expert) were proposed. Conclusions: The FM experts supported the program and recognized its usefulness. To implement the program within the primary healthcare system, the program needs to be adjusted to accommodate professionals’ and patients’ reality, and institutional health policies must be improved by providing training on FM to the healthcare community.

## 1. Introduction

Fibromyalgia (FM) is a central sensitivity syndrome that causes widespread pain, insomnia, fatigue, mental health disorders and cognitive dysfunction [1]. It occurs worldwide with an estimated total prevalence in the general population of 1.78%, but it disproportionately affects women (3.98%) [2].

In recent years, our knowledge of FM has grown with respect both to the diagnostic criteria and pathophysiology and to the development of therapeutic approaches. Nowadays, multimodal and multidisciplinary treatment is considered the gold standard for treating FM, although it should also be combined with individual follow-up [3]. Based on the evidence, the 2017 Eular consensus [4] recommended a non-pharmacological multicomponent approach based on health education, physical exercise and cognitive–behavioral therapy. Some recent interventions that encompass these components and others, such as complementary medicine, have led to some improvements in the quality of life and symptom control of FM patients [5,6,7,8,9].

Since 2016, the Department of Health of Catalonia (Spain) has promoted the accreditation of Units Specialized in Central Sensitivity Syndromes (USCSSs), which are now providing multidisciplinary healthcare to FM patients as part of the public health system [10]. Groups working with FM patients and those who provide clinical assistance are considering whether primary care (PC) can offer these types of multicomponent treatments, given its accessibility and continuity of care and the cost-utility of such programs [11,12]. It is essential that the programs implemented be shaped according to the vision, expectations and experiences of the study population and the professionals involved in order to maximize their benefits [13,14].

The current qualitative study is part of a mixed-method study (ClinicalTrials.gov: NCT04049006) that aims to analyze the effectiveness, cost-utility and benefits of a 12-week multicomponent therapy program based on health education, physical exercise and cognitive–behavioral therapy in terms of the quality of life, functional impact, mood and pain of people who suffer from FM and who attend primary care centers (PCCs) of the Catalan Institute of Health (CIH) [15]. The qualitative study protocol has been previously published [16].

In the work reported in the present article, we aim to evaluate professionals’ perceptions and experiences of the program as well as the program’s usefulness and the barriers to its implementation. The professionals who participated in our program had been trained to become a group of FM experts working in each of the 11 PC teams of the CIH in the Terres de l’Ebre region. As far as we know, few studies have considered professionals’ experiences of FM programs with a view to redesigning them and the aim of promoting the implementation of programs that better match the needs and accommodate the opinions of these stakeholders.

## 2. Materials and Methods

### 2.1. Design

A qualitative, descriptive study based on focus groups (FGs) was performed. A pragmatic and utilitarian approach was used with the aim of conducting a useful, ethical, accurate and practical evaluation [17]. FGs were selected as the methodology to allow for discussion among small groups of the professionals who participated in the program and comparison of their responses that ‘focus’ on the relevant issues [18].

A semi-structured interview guide (Appendix A) was developed to conduct the FGs. It consisted of the following headings: experience as an FM expert in the program, including aspects of the doctor–patient relationship; usefulness and limitations of the face-to-face training and counselling received during the program by the USCSS; usefulness of the program and adequacy of the content and format (place, duration, time) of group sessions; views of the FM experts regarding how the patients had experienced the program; and areas of the program that require strengthening and opinions about following the program as part of PC. The research team was composed of researchers in clinical practice, public health and social sciences.

### 2.2. Professionals’ Training and Attributions in the Program

The FM experts were trained to perform a multicomponent therapy program based on health education, physical exercise and cognitive–behavioral therapy. Details of the program were previously published [15]. They received initial training in a face-to-face course organized by the USCSS, a multidisciplinary part-time team of professionals consisting of a nurse, psychologist, physiotherapist, general practitioner, rheumatologist and external experts. The training covered concepts of central sensitivity syndromes (CSCs) and FM, how to conduct a motivational interview, how to lead a group, the neuroscientific concept of pain, relaxation techniques and specific aspects of the content of the program components (physical exercise; health education about pain, diet, sexuality, etc.; cognitive behavioral therapy). In addition, they received training to manage follow-up tests, to record their results in the program’s computer application and to evaluate the indicators of evolution. Annual training was provided beyond the initial training with 1–2 sessions per year, each with a duration of 10–20 h, and all imparted between 2016 and 2021. The training was based on a continuous evaluation with a participatory dynamic and was accredited by the Training Unit of the Territorial Management of the CIH in the Terres de l’Ebre. A total of 19 FM experts were trained inside the program. The first wave of FM experts also helped co-design the session content that made up the program.

Once trained, the FM experts’ duties were to invite the patients to participate in the program, prepare and lead groups in PCCs, ensure that the patients completed the tests and record the test results at the beginning, at the end, and during follow-up. In addition, they provided training and information to fellow health professionals in their PC team. The USCSS team also carried out counselling that involved supporting the FM experts in carrying out all their duties that were part of the program, actively participating in the group sessions (in the cases of the psychologist, nurse, physiotherapist, and general practitioner) and advising on the management of complex patients.

### 2.3. Study Setting and Participants

Two FGs were conducted with all 12 active professionals involved in the program. These professionals were previously trained as FM experts and were involved in the program at the time of the FGs. The FGs each comprised six participants and were conducted in two PCCs in Tortosa, Spain. Eleven of the 12 professionals were female with a mean age of 54 (±9.90) years. Regarding the professional profile, ten were nurses, and three were general practitioners (GPs) with 2–5 years of experience as FM experts (Table 1). Twenty-five multicomponent groups were conducted between 2016 and 2021. The FM experts each participated in an average of 2–3 groups. All professionals were invited by email to participate in the FG, and to avoid absenteeism, a reminder email was sent 48 h before each scheduled FG.

### 2.4. Data Collection

The two FGs were performed on 16 June and 26 July 2021 and were held in a room of a PCC. The FGs lasted approximately 60 min each and were conducted by a moderator (AQG) and an observer (external professional) who took field notes during the sessions. The moderator belonged to the biomedical field (a PhD) and had training and experience in qualitative research, while the observer was a PC nurse. Both the moderator and observer were experienced in moderating and acting as an observer in FGs, respectively. They were not involved in the intervention phase of the program nor in the professionals’ training. The data were collected as digital audio recordings prior to written signed informed consent. The FGs were manually transcribed verbatim by an external specialist and anonymized to guarantee the participants’ anonymity.

### 2.5. Data Analysis

Three members of the research group participated in the triangulations, which consisted of repeatedly reading the text corpus and the observer’s field notes and independently performing a flexible, iterative thematic content analysis [19]. The method of thematic content analysis was chosen because it consists of analyzing the contents expressed in a direct way (“what is said”) and interpreting its meaning [20]. Two triangulations were performed on separate days. The first, performed with the transcription of one of the FGs, consisted of the analysis of preliminary analytical intuitions, the comparison of the researchers’ perspectives and discussion and agreement of codes and categories (by domains). These data were collated in a single document. For the second triangulation, the other FG was analyzed, and the document with the initial codes and categories was updated with new data by each researcher. The analysis performed by each researcher was compared and discussed in order to arrive at a consensual final document featuring the most prominent domains, emerging themes and representative quotations. Data saturation was taken to have occurred when no further relevant insights could be identified. No software was used to manage the qualitative data.

### 2.6. Trustworthiness

In the present study, we followed the trustworthiness criteria defined by Lincoln and Guba [21] and Korstjens and Moser [22]. To ensure credibility, a strategy of triangulation was adopted, as described in the previous section. A member-check strategy was carried out at the end of each FG in which the moderator summarized the main aspects reported by the participants to ensure the reports would be correctly understood and interpreted. The transcripts were not sent to the participants. A broad description of the context, setting, participants’ characteristics and the research process is provided to satisfy transferability criteria.

In terms of dependability, despite the absence of an external audit trail, the research steps were documented throughout the study by one of the researchers, as described earlier in the Methods. The confirmability criterion was met by including the most representative literal quotations and researchers’ interpretations. The researchers endeavored to maintain a reflective attitude throughout all phases of the study. Finally, the study followed the Standards for Reporting Qualitative Research (File S1) [23].

## 3. Results

The results were organized into four key domains according to the interview guide in order to focus clearly on the study objectives. The first domain comprises two themes related to the training undertaken before the program began and the counselling provided by USCSS staff within the framework of the program. The second domain, comprising three themes, covered aspects of the experience of the co-design and usefulness of the program, adequacy of the format of the group sessions and aspects of content. The third domain consists of three themes that described the experience of the FM experts of the program with particular emphasis on personal barriers to FM patient care, the adaptation of the program during the COVID-19 pandemic and barriers to program implementation in PC with proposals for overcoming them. The final domain comprises two themes related to the opinion of the FM experts about the patients’ experiences of the program, including their view on barriers to patient participation in and full enjoyment of the program (Table 2).

### 3.1. Domain 1: Training and Counselling within the Framework of the Program

#### 3.1.1. Training as Motivation

It was notable that the training within the program was generally highly valued by the professionals. They felt that they personally benefitted from the training received and the knowledge gained; these were more valuable to them than financial remuneration.

“…this gives me more knowledge, I already feel rewarded”.

(nurse, P8b)

The professionals positively valued learning about group communication techniques, how to lead groups (especially those made up of “complicated” patients, such as those with FM) as well as techniques with which to address the different components (psychological, health and physical education) of the program. Regarding possible improvements, they commented that continuous training should always be carried out (“every year”, according to one of the participants).

#### 3.1.2. Continuous Counselling

Regarding the counselling by the professionals of the USCSS, the participants expressed general satisfaction with the support and reported good accessibility when counselling was desired. On the other hand, some professionals disagreed and highlighted the need for more and continuous support from the USCSS staff. They also identified a need for more support during the first group they led after their training. Although the program was focused on FM patients, the FM experts also would have liked to obtain counselling about dealing with chronic fatigue in their patients, probably because this syndrome is frequently encountered in people also diagnosed with FM.

“…in the unit (the USCSS), more time should be available to give us support… I think it should be full time, not just three days…”.

(nurse, P10b)

### 3.2. Domain 2: Usefulness of the Program and Adequacy of the Content and Format of Group Sessions

#### 3.2.1. Co-designing Experience and Overall Program Assessment

Regarding the co-creation of the guide for implementing the program and conducting the group sessions, the professionals were of the opinion that co-designing the content with professionals from the USCSS was a positive experience, although some of them considered the final format to be unattractive and outdated.

The professionals believed that the program improved over time and was useful. They considered that the adoption of a multidisciplinary approach was beneficial to FM patients, since it promoted a more integral treatment. All the topics covered by the program were considered timely and necessary.

“It has been a gain to incorporate the physiotherapist and the psychologist into the project, and that it has been multidisciplinary. Because I think that, in this way, you cover much more and a more integral therapy is carried out”.

(nurse, P10b)

#### 3.2.2. Program Format Considerations

The number of sessions (twelve) was generally considered to be adequate, although participants considered that this was insufficient time to cover all the content. The professionals proposed changing the organization of the sessions so that there would be less content over each 2 h session in order to avoid the patients becoming tired. Some participants suggested that the relaxation session should be longer, since the patients themselves stayed longer than the scheduled duration of the session. The schedule was adapted to the preferences of all the patients in each group as far as possible, but it was difficult to find a good common schedule that suited everybody. The PCC was found to be an adequate space, especially because it is the place where the professionals work; however, one participant suggested that the most important consideration is for the place to be comfortable with little noise and a high degree of privacy. According to the professionals, the patients also had reservations about the lack of confidentiality in the PCC because people in their area usually know each other, especially in villages.

“…there are some (patients) who don’t want to be seen going to groups… sometimes in the health center you run into a lot of people”.

(nurse, P10b)

#### 3.2.3. Aspects of the Content of Sessions

The participants agreed that the topics addressed in the health education sessions were relevant. They appreciated the content of the topic of pain, especially the session on the neuroscientific perspective on pain, and the themes of nutrition and management of rest. Regarding the sexuality sessions, the FM experts highlighted the difficulty patients had talking about sexual matters, which appeared to them to be a matter of taboo. The professionals suggested that it is also difficult for the participants to address this subject with their partner. Effort was expended adapting the physical exercise and relaxation sessions, taking into account the limitations of each patient.

“… I always tell them: you don’t all have to do it the same (physical exercise)… there are people who cannot do a long relaxation”.

(nurse, P10b)

However, some professionals thought that too little time was devoted to physical exercise, and for this reason, they felt that the patients did not place adequate importance on it. One of the professionals proposed the development of a video of the exercises as a means of overcoming this.

The cognitive–behavioral therapy was considered an excellent experience due to the contribution of the psychologist who led the sessions, the quality of the content and the structure and duration of the sessions. In their opinion, the psychological sessions were the ones patients most needed, and those that did help them change their approach. They also recognized that the patients wanted it: ‘The patients are waiting for it’. One professional suggested that, in reality, this therapy was “new” for patients only because pharmacological treatment and physical exercises were not.

“…since the medication does not go very well for them… the exercises, some can do but some can’t… the behavioral part was what changed them, I think”.

(nurse, P3a)

### 3.3. Domain 3: Experience as an FM Expert and Proposals for Overcoming Barriers

#### 3.3.1. Facing the “Unknown” and Breaking Personal Barriers

The experience of the professionals as FM experts in the program was generally good. They reported a positive change in their expectations, since some of them, initially, did not want to take on the role of FM expert or had been obliged to do so. Further, they did not feel prepared to deal with patients they could not easily understand.

“…they asked me to be an FM expert, I said no, the first answer was: “no, I don’t understand them and it’s impossible””.

(nurse, P7b)

The FM experts had a very positive experience of leading groups of patients with FM. It made them feel closer to their patients, enabling them to understand them better.

“Neither did I like them, nor did I generate empathy. After that (starting to lead the group), this relationship began to improve… you relax, you get to know them, you get to know the disease, you go in and you no longer put up that barrier when it comes to relating”.

(GP, P12b)

There was recognition of the good work performed, whereby the professionals felt they had benefitted in terms of human values, satisfaction, feeling useful and learning and growing as professionals. They commented that a bond was created with the patients, and some friendships were even formed.

“…I have patient friends who are no longer patients… with the younger ones we go for a walk some Sundays”.

(nurse, P11b)

#### 3.3.2. “Lessons Learnt” from the COVID-19 Era

The participants commented on the adaptation of the sessions that was essential during the period of the COVID-19 pandemic, whereby four groups had adopted a mixed format with some sessions held online and others face-to-face. One participant commented that further improvements to this way of working were needed to overcome technical problems and to foster greater confidence in the approach. This participant considered it important that the session with the psychologist be held face-to-face because she believed that patients benefit more from that format. Another participant argued that physical exercise sessions should not be conducted online because of the risk of injury. A third participant believed the sessions should consist of a mixture of face-to-face and online meetings.

#### 3.3.3. Barriers to Program Implementation in PC and Proposals to Overcome Them

The professionals encountered some difficulties. They reported that they were overloaded and under considerable pressure from their work as an FM expert. They highlighted the difficulty of reconciling their duty to assist patients with fulfilling the timetables of the groups, organizing the sessions and setting aside time to record the test results in the program’s computer-based application. Some of them felt overwhelmed because they had to ensure that their patients completed the follow-up tests at the beginning and end of the group sessions and 6 and 12 months later.

“…create the groups, between patient and patient… this overloaded me”.

(nurse, P3a)

They commented on feeling alone and being misunderstood by peers who were not involved in the program and who did not appreciate its value and the time that had to be devoted to running the groups.

“…it seems as if the schedule you are investing in is not important, what is important is the other schedule (that of the consultation)”.

(nurse, P7b)

They were convinced that all professionals should be committed to working with FM patients.

“…the other (professionals) must be involved because the patient with fibromyalgia is not a patient of the FM expert, she/he is a patient of everyone”.

(nurse, P4a)

One participant explained that some of the GPs’ attitudes to the program were a reflection of what society in general thinks about FM:

“…It’s true that fibromyalgia, at the level of society, is frowned upon and people don’t understand them (the patients), neither do the family members… and that’s what costs the most, sometimes, at a professional level, to “get into” in this project”.

(nurse, P10b)

Considering the outlook for the program, the professionals believed that the accessibility of PC would favor its future implementation. However, the role of the FM expert must first be well-defined, and the program needs to be well-structured and supported by adequate human resources as required. Some professionals believed that it would help other healthcare professionals to accept the reality of FM if the diagnosis and follow-up of these patients were considered as quality-of-care indicators by the professionals. They were also of the opinion that holding more sessions to inform healthcare professionals could facilitate their acceptance of and commitment to the program. It was proposed that other categories of professionals, such as administrative staff, should be involved in the program.

“…just as a professional knows how to care for a diabetic or a hypertense patient, they should know how to care for a person with fibromyalgia”.

(nurse, P3a)

### 3.4. Domain 4: Views of FM Experts about Patients’ Experiences of the Program

#### 3.4.1. Confronting the Disease in a Different Way

The professionals perceived that the patients had benefited from their participation in the program. They remarked that, in general, the patients became involved, learned valuable information and were satisfied with their experience of the intervention. Some patients took the program very seriously and performed the exercises at home that they had learned in the sessions. However, they were aware that, although the health status of some patients improved, that of others did not.

The group intervention helped patients approach the disease in a different way. The professionals realized that the group was a safe place for the patients that gave them the confidence to share intimate aspects of their personal life with other people, even though, as they pointed out, this was not one of the goals of the groups. The professionals took the view that the patients realized that other people also suffer from FM, which led them to feel that, finally, they were understood.

“At home they (relatives) do not understand them and there (in the group) everyone understands them…, the fact that the treatment is in a group is more positive”.

(nurse, P2a)

They also felt that the patients were grateful for having received help from the program and for having been “heard”.

“Even if they have relapsed, they know that you have listened to them, you have attended to them and you have paid attention to them”.

(nurse, P3a)

Some of the professionals received feedback from the patients that they would have liked to follow-up after the groups had ended. The professionals reported an indirectly beneficial effect whereby some patients maintained their relationships even after the groups had ceased to meet.

“…they related a lot. To this day they are still friends, they always write to each other in the WhatsApp group”.

(nurse, P5a)

#### 3.4.2. Barriers to Patient Participation in and Full Enjoyment of the Program

The professionals reported difficulties in rural areas to conduct groups due to the dispersed population, which involved forming groups with patients from different rural areas. Some patients had problems with travelling to attend sessions, especially if they attended a different rural health center from the one where they came to take part in these sessions:

“…in rural areas it costs a lot to form a group, because there is a lot of dispersion, they are usually women who don’t have a car”.

(nurse, P2a)

The professionals commented that the patients were asked to do many things at home after the sessions (e.g., relaxation, physical exercise, etc.) but that some of them failed to achieve this and, therefore, had to be guided towards adapting the activities to fit in with their personal circumstances:

“…we say: “you don’t have to collapse, you have to try to see which are the best (activities, exercises) and alternate, you don’t have to do everything on the same day””.

(nurse, P10b)

## 4. Discussion

The findings of this qualitative study indicate that the multicomponent program for FM patients developed in the primary healthcare system was positively valued by the FM experts. These professionals were members of a group of nurses and GPs from the public PC of the Terres de l’Ebre region (south Catalonia, Spain) who were trained with the objective of becoming experts in their PC team not only to carry out the multicomponent program but also to act as a local consultant for health professionals. The training aimed to make FM treatment more locally available to patients, thereby improving the accessibility of services through the inherent characteristic of proximity of PC to the citizen [24]. Multicomponent treatments are currently considered the “gold standard” along with maintaining individualized care [3]. Recent quantitative studies are confirming the effectiveness of some multicomponent treatments for FM patients [6,8,25,26,27]. It is essential that strategies be tested to promote equality of care across different settings. In our case, the study area comprised small- and medium-sized urban centers and large rural areas. The recent incorporation of new specialists, such as psychologists and physiotherapists, into the PC teams of the CIH [28,29] can boost the activities of multidisciplinary therapeutic groups throughout our territory.

The training developed as part of the program was well valued by the FM experts as it provided a good opportunity for acquiring knowledge. There were reports of personal satisfaction, although they highlighted the need for more continual training and counselling by the USCSS professionals. The need for this continuity is paradoxical since training was offered at least annually during the program. We believe that this perception could be a reflection of the desire to feel safer and/or more appreciated in their new role. These perceptions could have arisen for two reasons: (1) the profile is new to the institution, and the participants, therefore, felt their role needed to be better defined; and (2) the lack of acceptance of the role among professional colleagues generates discomfort in the relationship with their peers.

The professionals considered that the program was useful for promoting integral treatment through a multidisciplinary approach, resulting in an improvement in the health of some patients. The PCC was judged to be adequate for the sessions, although it was pointed out that patients from villages regarded the PCC as a setting with little confidentiality due to the risk of being seen by their neighbors. This discomfort probably arises from the social stigma related to FM, a lived experience frequently expressed by FM patients in qualitative studies [30,31]. In relation to the format of the sessions, the professionals suggested that some improvements should be made whereby less content was provided during each session to avoid tiring the FM patients. As for the content of the sessions, the professionals identified pain, nutrition and management of rest as important topics, although cognitive–behavioral therapy was identified as the most needed and preferred by the patients. Psychological care is frequently demanded by FM patients in the health system [32] due to the necessity to seek strategies to cope with this syndrome on a day-to-day basis. Briones-Vozmediano et al. [33] concluded in their qualitative study of professionals and FM patients in Spain that the public health system does not generally offer well-timed care and that patients face difficulties finding a solution to their health problems within it. On the other hand, as previously mentioned, the recent incorporation of new health specialties in PC teams marks a promising change for the better in the CIH’s PC and could represent a good opportunity to strengthen the programs coordinated by the USCSS units throughout Catalonia, Spain.

Although attempts were made to adapt the physical exercise sessions to the FM patients’ individual circumstances, the professionals noted that the patients attached little importance to them, probably because the time available for this activity was too short. To tackle this, the professionals proposed that a video be produced that would encourage better patient adherence to the exercise regime in their own home. Current studies indicate that undertaking therapeutic exercise and ensuring that the recommended time is dedicated to the activity in combination with behavioral activation are key to improving FM symptoms [34,35].

In relation to the sessions addressing sexuality, the professionals concluded that this topic was considered taboo by the patients, since they noted the difficulties that patients had talking about it. However, it is possible that a session that only lasted one hour did not create a suitably intimate environment for sharing experiences. In a qualitative study of perceptions of sexuality of women with FM, the authors reported that guilt and fear had affected the patients’ sexual relationships, particularly with regard to pleasing their partner, although women stated that sex was important to their quality of life and identity [36].

Some participants found their experience as an FM expert in the program to be professionally challenging. When the program began, some of them did not want to be an FM expert or did not feel qualified to care for the patients, probably because they held a stereotypical view of them as “difficult patients” who they would not be able to understand well enough. However, some of them described how their initial feeling of discomfort with the new role changed towards one of satisfaction with their work that, in some cases, even led to the formation of doctor–patient friendships. Briones-Vozmediano et al. [33] described reports of tense relationships between professionals and FM patients whereby the former felt their professionalism was challenged and the latter expressed dissatisfaction towards the health system.

During the period of restrictions during the COVID-19 pandemic, the program had to be restructured with some sessions being held online. The professionals identified difficulties with adapting to this new format, and a few considered that some of the sessions, in particular those on psychological aspects and physical exercise, should always be conducted face-to-face to ensure the greatest benefit to patients and, in the case of exercise, to avoid injuries.

The professionals identified significant barriers to implementing the program in their particular settings. Overload is a frequently reported barrier in PC studies [37] and was identified here by the FM experts. In our study, the professionals who were assigned to the new role had to reconcile completing their new tasks with continuing to provide clinical care. However, the number of hours allocated to this new professional profile varied considerably among the different PC teams within the same territory, depending on the resources available locally. The feeling of overload was widespread among the FM experts, irrespective of the time and resources available to them. It is important to note that part of the feeling of overload was due to the large number of follow-up tests that were carried out only as part of the clinical trial itself and that would not be required as part of a habitual care program. Another barrier stems from the lack of understanding on the part of their fellow workers and the feeling of being judged for investing working hours in treating a syndrome that is still not completely accepted as being a genuine entity by the medical community. These aspects led to feelings of loneliness and of being misunderstood. At the end of their experience with the program, all FM experts were clear that all professionals should be committed to the care of FM patients. Societal prejudices were understood to be present in the lived experience of patients in PC. In a study about the social construction of FM, the health care professionals interviewed in Spain regarded FM as being a condition affecting women and admitted that they assigned negative stereotypes to FM patients and that prejudices existed among the collective of health professionals [38].

To improve the program, the professionals proposed that the role of the FM expert should be better delineated, and a well-defined program should be established that provides the necessary human resources in a timely manner to ensure its successful deployment. The training of health professionals regarding FM and the creation of quality indicators of professional care were also considered worthwhile. Briones-Vozmediano [38] also proposed training for healthcare personnel not only to improve knowledge and competencies but also to provide adequate support, to increase sensitivity to patients’ needs and to raise awareness of the tendency to stereotype female FM patients.

With respect to the patients’ experience of the program, the professionals observed their satisfaction, the improved health in some but not all cases and the willingness to participate in follow-up activities after the group sessions had ended. The professionals detected a “group effect” whereby people with the same health problem could meet as a group in a place where they felt confident about sharing their experiences and, at last, be listened to by health professionals and be understood by fellow sufferers. These aspects were corroborated in the FGs with patients from the present study [39] and in other programs for FM patients [40]. The relationships generated during the program were so strong that some of the patients remained in contact with each other through a WhatsApp group. The barriers to participation included the problems experienced by some patients from rural areas to travel to the health center where sessions were held, especially as a result of transportation difficulties in more remote geographical areas. Also, during the implementation of the program by the PC teams, some of the groups had to be made up of patients from different rural areas, which proved difficult for some patients to go to the session site. Another barrier arose from the requirement to offer a program that every patient would enjoy, since each FM patient has her/his own limitations. Individualized adaptation of the content of the sessions is essential to the success of the program.

From the patients’ point of view [39,41], the program was positively valued for its holistic approach, which helped them gain knowledge and acceptance of the disease. The patients also highlighted the improvements in their coping skills and ability to self-manage their symptoms, the health benefits that include the alleviation of suffering and the promotion of the legitimacy of FM. To improve the program, they proposed that some of its topics should be tackled in greater depth, the duration of the intervention should be extended and additional psychological support should be offered and information provided to relatives.

### Limitations

In the present study, we analyzed the discourse of the FM experts trained within the framework of the program. However, it is important to bear in mind that the FM experts were not all trained at the same time and joined the program over the course of several years. As only active professionals participated in the FGs, it may have prevented other themes from being identified during the discussion, especially those related to the beginning of the program and experienced by the first wave of trained professionals. In addition, the number of FM experts differed across PC teams and over time (one or three per PC team). These aspects are likely to have influenced the perceptions of the participants. The professionals’ feelings of work overload in the PC teams may have varied according to the number of FM experts, and their sense of security in the new role may have differed depending on how recently they had performed their training. We note the remarkable preponderance of women among the participants, a situation that reflects the predominance of healthcare professional women in PCCs in Spain [37]. The COVID-19 pandemic represented an important barrier with the program activities suddenly reduced. However, we observed that this period did not affect the good overall assessment of the program by the professionals and patients; rather, the professionals’ perceptions during the pandemic period add value to their overall experience into the program.

## 5. Conclusions

The present study demonstrates the acceptability among FM experts of the multicomponent group intervention program for FM patients in the primary healthcare system of Spain. Although the professionals acknowledged the usefulness of the program, we identified some barriers to its implementation, notably work overload and the lack of acknowledgement of the program’s importance among their peers. In the future, to implement the program within the primary healthcare system, it must be adjusted to concur with professionals’ and patients’ views. The new professional role of FM experts needs to be promoted. Institutional health policies require improvement by providing the healthcare community with training on FM.

## Figures and Tables

**Table 1:** Characteristics of the participants in the focus groups.

Code	Gender	Age	Profession	Years Employed	Years as Fm Expert
P1a	female	61	GP	33	5
P2a	female	48	nurse	27	2
P3a	female	47	nurse	25	4
P4a	female	52	nurse	17	5
P5a	female	62	nurse	35	4
P6a	male	64	GP	17	4
P7b	female	53	nurse	32	5
P8b	female	55	nurse	28	3
P9b	female	54	nurse	32	5
P10b	female	64	nurse	43	2
P11b	female	44	nurse	22	3
P12b	female	47	GP	17	4

P: professional; a: focus group a; b: focus group b; GP: general practitioner; FM: fibromyalgia.

**Table 2:** Domains and themes.

Domains	Themes
1. Training and counselling within the framework of the program	Training as motivation
	Continuous counselling
2. Usefulness of the program and adequacy of the content and format of group sessions	Co-designing experience and overall program assessment
	Program format considerations
	Aspects of the content of sessions
3. Experience as an FM expert and proposals for overcoming barriers	Facing the “unknown” and breaking personal barriers
	“Lessons learnt” from the COVID-19 era
	Barriers to program implementation in PC and proposals to overcome them
4. Views of FM experts about patients’ experiences of the program	Confronting the disease in a different way
	Barriers to patient participation in and full enjoyment of the program

## Data Availability

The datasets used and analyzed during this study are available from the corresponding author upon reasonable request.

## Supplementary Materials

The following supporting information can be downloaded at: https://www.mdpi.com/article/10.3390/healthcare12010017/s1, File S1: Reporting checklist: Standards for Reporting Qualitative Research (SRQR) [23].

## Appendix A

**Focus Group Interview Guide**

A. Greetings and presentation of the research team.
B. General information on the focus group (FG): objectives and dynamics.
C. Explanation of the ethical aspects.
D. Individual presentation of the informants.
E. FG questions:

*Experiences as a fibromyalgia (FM) expert*

1. What was your experience as FM experts within the program?
2. What are the positive aspects of the training as an FM expert and the advice received by the Unit Specialized in Central Sensitivity Syndromes (USCSS) during the program?
3. What are the negative aspects of the training as an FM expert and the advice received by the USCSS during the program?

*Aspects of the program/content of the group sessions and technical aspects*

4. What did you think of the group program for the treatment of fibromyalgia in general? (useful aspects)
5. What did you think of the content of the group health education sessions carried out as part of the program?
6. What did you think of the content of the group physical exercise sessions carried out as part of the program?
7. What did you think of the content of the group psychotherapy sessions carried out as part of the program?
8. What did you think of the place where the group sessions were held (health centers), their duration and scheduling?
9. What was your experience with fibromyalgia patients during the program? Do you want to comment on anything relevant to the doctor–patient relationship within the program?

*Opinions about the patients’ experience in the program*

10. What is your opinion about the degree of patient satisfaction with the program?

*Aspects of the program that could be improved and program continuity*

11. What aspects of the current group program should be improved?
12. What do you think about the continuation of the program in primary care?

*Contributions*

13. Would you like to comment on any other point or topic that has not been mentioned?

F. Ending of the FG:
  - Summary of the informants’ contributions.
  - Acknowledgement and thanks.
